# The FReedom from Ischemic Events - New Dimensions for Survival (FRIENDS) registry: design of a prospective cohort study of patients with advanced peripheral artery disease

**DOI:** 10.1186/1471-2261-13-120

**Published:** 2013-12-19

**Authors:** Hong H Keo, Sue Duval, Iris Baumgartner, Niki C Oldenburg, Michael R Jaff, JoAnne Goldman, James M Peacock, Alexander S Tretinyak, Timothy D Henry, Russell V Luepker, Alan T Hirsch

**Affiliations:** 1 Division of Angiology, Kantonsspital Aarau AG, Aarau, Switzerland; 2 Vascular Medicine Program, Lillehei Heart Institute and Cardiovascular Division, University of Minnesota Medical School, Minneapolis, MN, USA; 3 Swiss Cardiovascular Center, Division of Angiology, University Hospital Bern, Bern, Switzerland; 4 Massachusetts General Hospital and Harvard Medical School, Boston, MA, USA; 5 Minneapolis Heart Institute Foundation at Abbott Northwestern Hospital, Minneapolis, MN, USA; 6 Minnesota Department of Health, Heart Disease and Stroke Prevention Unit, Saint Paul, MN, USA; 7 Division of Epidemiology and Community Health, University of Minnesota School of Public Health, Minneapolis, MN, USA

**Keywords:** Peripheral artery disease, Amputation, Atherosclerosis, Health service research, Outcomes research

## Abstract

**Background:**

Advanced lower extremity peripheral artery disease (PAD), whether presenting as acute limb ischemia (ALI) or chronic critical limb ischemia (CLI), is associated with high rates of cardiovascular ischemic events, amputation, and death. Past research has focused on strategies of revascularization, but few data are available that prospectively evaluate the impact of key process of care factors (spanning pre-admission, acute hospitalization, and post-discharge) that might contribute to improving short and long-term health outcomes.

**Methods/Design:**

The FRIENDS registry is designed to prospectively evaluate a range of patient and health system care delivery factors that might serve as future targets for efforts to improve limb and systemic outcomes for patients with ALI or CLI. This hypothesis-driven registry was designed to evaluate the contributions of: (i) pre-hospital limb ischemia symptom duration, (ii) use of leg revascularization strategies, and (iii) use of risk-reduction pharmacotherapies, as pre-specified factors that may affect amputation-free survival. Sequential patients would be included at an index “vascular specialist-defined” ALI or CLI episode, and patients excluded only for non-vascular etiologies of limb threat. Data including baseline demographics, functional status, co-morbidities, pre-hospital time segments, and use of medical therapies; hospital-based use of revascularization strategies, time segments, and pharmacotherapies; and rates of systemic ischemic events (e.g., myocardial infarction, stroke, hospitalization, and death) and limb ischemic events (e.g., hospitalization for revascularization or amputation) will be recorded during a minimum of one year follow-up.

**Discussion:**

The FRIENDS registry is designed to evaluate the potential impact of key factors that may contribute to adverse outcomes for patients with ALI or CLI. Definition of new “health system-based” therapeutic targets could then become the focus of future interventional clinical trials for individuals with advanced PAD.

## Background

Advanced lower extremity peripheral artery disease (PAD) represents a relatively uncommon, but exceptionally high-risk, manifestation of systemic atherosclerosis. Both traditional PAD classification systems and recent evidence-based PAD care guidelines have emphasized the potential benefit of more consistent case classification of advanced PAD into two distinct clinical syndromes, denoted as acute limb ischemia (ALI) or chronic critical limb ischemia (CLI). Both PAD syndromes represent manifestations of a severe compromise of arterial blood flow to the affected lower extremity, with the acuity of onset of ischemia and specific clinical signs used to distinguish between these syndromes [1]. In contrast to comparable acute coronary disease syndromes (such as the acute coronary syndromes [ACS] of non-ST segment myocardial infarction (MI) and ST-elevation MI), the epidemiology of ALI and CLI is much less well defined in the absence of population-based surveillance. Current evidence suggests an incidence of approximately 430 to 1160 cases per million individuals annually [2-4]. Although ALI and CLI represent the primary etiology of most lower-limb amputations [5] and are associated with high short-term rates of systemic and limb ischemic events [6,7], uncertainty regarding the incidence, prevalence, and outcomes of the ALI and CLI syndromes is, in part, based on the lack of a uniform and applicable definition used in either field studies or in clinical practice outside of specialized vascular centers.

Improvements in coronary ischemic syndrome health outcomes have been facilitated by consistent use of ACS definitions and subsequent evaluation of health system factors that contribute to cardiovascular risk. For example, improved outcomes for patients with ST-elevation MI and acute stroke were achieved by evaluation of methods to shorten end-organ ischemia time (including patient recognition of heart attack symptoms and creation of expedited referral systems) and this knowledge has been successfully encompassed in cardiac and stroke care guidelines [8,9]. In contrast, little data exist to define the contribution of limb ischemia time to adverse limb or systemic outcomes for individuals with ALI and CLI. Similarly, whereas the impact of pharmacological and behavioral risk-reduction interventions (e.g., use of antiplatelet and statin medications) is well-defined for patients with ACS, a knowledge gap exists regarding the potential impact of these systemic treatments after successful leg arterial revascularization. This knowledge gap is central for patients with advanced PAD as recent data document that both endovascular and open surgical approaches, while effective in improving limb outcomes for individuals with CLI [10], are still associated with very high rates of heart attack, stroke, and cardiovascular death within one year of an index CLI presentation.

Randomized clinical trials (RCTs) provide the most robust methodological approach to determining the efficacy, harm, and net impact of potential treatments for individuals with advanced PAD. Nevertheless, recent experience has demonstrated the challenges and costs associated with recruitment of individuals with ALI and CLI into prospective RCTs. As for coronary disease or heart failure clinical research, there are potential advantages to the study of outcome determinants from a prospective disease registry. These advantages include: enrollment of the full patient cohort with minimal exclusion criteria, the use of a multicenter design that is often more broadly representative of the ALI and CLI population than might be recruited from specialized centers that participate in sponsored RCTs, the potential to capture longer-term outcomes data, the ability to *pre hoc* define outcome determinants (e.g., pre-hospital leg ischemia time) that are not easily evaluated in a RCT context, and often the ability to collect such data at lower cost [11-13].

In this context, specific knowledge gaps regarding determinants of adverse health outcomes for individuals with ALI and CLI were used to design a hypothesis-driven clinical registry. The primary aim of the FRIENDS registry is to evaluate a pre-defined set of patient and health system factors on one-year rates of systemic and limb ischemic events, and thus on amputation-free survival. These factors included: (i) pre-hospital limb ischemia symptom duration, (ii) use of leg revascularization strategies, and (iii) use of risk-reduction pharmacotherapies. Outcome events (rates of non-fatal MI, stroke, amputation, and death) will be collected at one month, six months, and one year. Secondary study aims are to assess: (a) the relative frequency and etiology of the ALI and CLI diagnoses in a large community-derived population, (b) the frequency of use of evidence-based risk-reduction medication in this high-risk population at baseline and during follow-up, (c) predictors of functional status, and (d) rates of systemic ischemic events. The FRIENDS registry structure was *pre hoc* designed to serve as a pilot study for future implementation of a multicenter registry to prospectively evaluate the impact of standards of care on health outcomes for all individuals with ALI and CLI.

## Methods/Design

### Study design

The registry is designed as a prospective cohort evaluation of individuals with advanced PAD, defined as inclusive of the ALI and CLI syndromes.

### Enrollment

We defined the index hospitalization as the first presentation for ALI or CLI, since a hospital admission serves as an easily identified site for detection of such cases. Use of an index hospitalization is also advantageous (as compared to recruitment from vascular specialty clinics or wound centers) since it defines a population in whom health care resource utilization is high and in which quality outcomes staff now exist to facilitate initial and subsequent outcomes data collection. Patient enrollment is intended to be sequential and fully inclusive, with exclusions limited to non-provision of informed consent that are documented by a screening log.

### Definition of advanced PAD syndromes

The registry was designed to utilize definitions of advanced PAD (ALI and CLI) that are encompassed in the intersocietal “ACC/AHA Guidelines for the Management of Patients with PAD” [1] and concordant TASC-II [2] PAD guidelines. As such, ALI is clinically defined by the admitting vascular specialist in patients who experience the clinical stigmata associated with acute ischemia and a potential threat to limb viability of less than or equal to 14 days in duration. Using this guideline-derived ALI case classification, enrollment of patients is achieved via documentation of acute limb ischemic symptoms and signs (the 6 “Ps”) (Table 1). CLI will be defined, per these PAD clinical care guidelines, in patients with Rutherford class 4, 5, or 6 manifestations such as ischemic rest pain, and minor or major tissue loss of greater than two weeks duration. At enrollment, patients with ALI and CLI will undergo evaluation of an objective pedal or toe pressure measurement when this is feasible pre-revascularization. Source documents have been created so that collection of these entry criteria, as applied by participating clinicians, will be uniformly obtained.

**Table 1:** Inclusion criteria: Ischemic symptoms and signs, and objective limb pressure measurements at enrollment

**For all patients: Clinical symptoms and signs**	**For ALI patients: Presence of any of the “6 Ps”**	**For all patients: Objective pressure measurements**
Ischemic rest pain	Pain	Pedal pressures
Ulcer	Pale	Great toe pressures
Gangrene	Pulseless	Ankle-brachial index
	Paresthesia	Toe-brachial index
	Paralysis	
	Poikilothermia	

*Abbreviations*: *ABI* Ankle-brachial index, *TBI* toe-brachial index.

### Use of source documents and data abstraction

This is a prospective registry, and as such all sequential eligible patients with ALI or CLI who are hospitalized (defined as requiring an index leg revascularization procedure or overnight stay) will be entered into the registry. Medical record documentation will be required immediately on admission (not retrospectively) for establishment of the presence of ALI or CLI. Vascular specialty and hospitalist staff will receive instruction on: (a) the clinical definitions of ALI and CLI, (b) how to complete a source document on patient admission that includes the time of onset of symptoms, and (c) the use of health system tools (e.g., standardized order sets) to facilitate prompt measurement of pedal or great toe pressures (Table 1).

A documented medical history of coronary artery disease (CAD) will consist of 1 or more of any of the following chart documented conditions or procedures: stable angina, previous myocardial infarction, history of percutaneous coronary intervention or history of coronary artery bypass graft surgery. Documented cerebrovascular disease (CVD) will be defined by a history of transient ischemic attack (TIA), ischemic or hemorrhagic stroke, carotid stenting or carotid endarterectomy. Documented prior PAD will be defined by 1 or more of the following criteria: symptomatic PAD defined by history of claudication, asymptomatic PAD documented in the medical records, or history of ALI or CLI.

Baseline height and weight will be obtained from the medical records, and body mass index (BMI) calculated. Patients will be considered overweight if they have a BMI of 25 kg/m^2^ to less than 30 kg/m^2^ or obese if they have a BMI ≥30 kg/m^2^. Atherosclerosis risk factors will be defined as present: if documented in the medical record, if the patient is receiving treatment for the risk factor at the time of study enrollment, or if there are supporting laboratory test data proximal to the index hospitalization. Diabetes will be considered present when there is chart documentation, regardless of type (I or II), or when use of an anti-diabetic medication or dietary intervention is documented. As well, the documentation of fasting blood glucose ≥7 mmol/l (126 mg/dl) will be considered evidence of diabetes when these laboratory data are available. Hypertension will be defined when chart documented or when there is evidence of treatment with any antihypertensive medication or behavioral intervention (e.g., use of calcium channel blockers, ACE-inhibitors, beta blockers, diuretics or diet/exercise). Dyslipidemia is considered present when there is chart documentation or use of any lipid-lowering or cholesterol modifying medication (e.g., statin, niacin, fibrates, or bile acid binding resin agents). Dyslipidemia is also considered to be present if, per the National Cholesterol Education Program (NCEP) guidelines, the total cholesterol concentration is >200 mg/dl (5.18 mmol/l), LDL concentration is >130 mg/dl (3.37 mmol/l), and/or HDL concentration is <40 mg/dl (1.04 mmol/l). Other criteria for establishment of dyslipidemia include a fasting triglyceride concentration of ≥200 mg/dl, or total cholesterol/HDL ratio ≥5.0. Smoking status is recorded as “current” if any cigarettes were used within one month of the encounter, “former” if smoking had ceased more than one month prior to the encounter, and “never” if there is no history of tobacco use. A family history of premature CAD consists of any chart documentation of a family history of premature CAD or any myocardial infarction (MI) in a first-degree relative (men <55 years or women <65 years). The etiology of ALI or CLI is coded from data obtained in the medical record.

Ambulatory status on admission and discharge will be obtained from patient self-report and supplemented by abstracted medical record reports of treating clinicians. There is at present no single well-accepted format for recording ambulatory status in individuals with ALI and CLI. Ambulatory status therefore will be categorized into five groups and assigned ordinal values of 1 to 5, with 5 representing the best, and 1 the lowest, ambulatory status. The highest class (5), “fully ambulatory”, is defined as the ability to independently ambulate and fully interact in the community. “Minimal ambulation” (4) is defined as ambulation limited to within the house/hospital, around the house/hospital, and to the mail box. “Ambulation with assistance” (3) is defined by ambulation requiring another person’s help for mobility. “Wheelchair bound” (2) is defined by dependency on a wheelchair for all mobility. “Bed bound” (1) is defined when the patient is not mobile at all, is fully restricted, and non-ambulatory. Ambulatory status at baseline is the level of function after onset of ALI or CLI and within one week prior to admission. Data will be abstracted by trained research staff and reviewed by vascular physicians.

All data will be collected via a standardized case report form and then entered into a password-protected database. Data will be collected consistent with the intersocietal “2012 ACCF/AHA/ACR/SCAI/SIR/STS/SVM/SVN Key Data Elements and Definition for Peripheral Atherosclerotic Vascular Disease” standards [14]. Outcome events will be reviewed and adjudicated by a clinical event committee consisting of individuals with expertise in vascular surgery and vascular medicine. The registry design has been approved by the local institutional review board at the University of Minnesota and Allina Health System. All included patients will provide written informed consent. Patients screened for entry who do not offer informed consent will be entered into a screening log.

### Key time segments

The symptom onset of an initial episode of ALI/CLI is defined by the patient’s self-report provided to the vascular specialist at hospital admission, is dictated into the encounter note, and this note (date and time) is later abstracted from the medical records. Vascular specialists (vascular surgeons, vascular medicine specialists, and interventional radiologists) are instructed to document the patient-reported index limb symptom onset within pre-specified time parameters: within one hour for patients presenting within 3 days, within one day for patients presenting within 3 to 30 days, and within one week for patients presenting greater than one month since symptom onset. For CLI patients who cannot state an exact time of leg symptom onset, it will be assumed that the onset time is 12 noon on the day specified. Inasmuch as ALI or CLI is known to recur (repeat episodes are common), the index leg symptom is arbitrarily defined as that which is temporally closest to the index hospitalization. This approach is similar to that used for ACS or stroke health services research.

While the pre-admission symptom duration serves as the primary pre-defined hypothesized predictor of advanced PAD health outcomes, a number of other clinical care time segments are considered of potential utility in evaluation of the efficiency of hospital-based PAD care, as it is known that CLI hospitalizations are lengthy (greater than seven days) and expensive [15]. The “admission to ABI/TBI” time segment is defined as the time from hospital arrival to initiation of the first hospital-based pedal or toe pressure measurement. The “admission to heparin” time is defined as the time from hospital arrival to the chart-documented initiation of a therapeutic intravenous heparin infusion for patients so treated. The “admission to vascular specialist at bedside” time segment is defined as the time from hospital arrival until the patient is evaluated by a vascular specialist, and this is set by the time of the dictated note. The “admission to revascularization” time segment is defined as the time from hospital arrival until the patient enters the operating room or catheterization laboratory, and includes only the use of an endovascular procedure, surgical procedure or primary amputation (medical treatment alone is not included in this definition). Hospital length of stay is defined as the number of days of hospitalization from admission to discharge.

#### List of pre-specified “key time segments” for patients with advanced peripheral artery disease

Symptom onset to admission

Admission to ABI/TBI measurement

Admission to IV heparin administration

Admission to vascular specialist at bedside

Admission to revascularization

Length of stay (days)

### Study outcomes

The study outcomes that will be evaluated are summarized in Table 2. The systemic ischemic risk clinical outcomes include: nonfatal MI, nonfatal stroke, cardiovascular (CV) death, all-cause death, and cardiovascular re-hospitalization event rates. The limb ischemic risk clinical outcomes include: ALI or CLI recurrence, minor or major amputation, and total amputation event rates. These individual outcomes will be used to derive a composite triple endpoint (defined as any nonfatal MI, stroke or amputation) and quadruple endpoint (defined as any non-fatal MI, stroke, amputation or death). As well, the relevant clinical outcome of “amputation-free survival” time will be calculated at one year.

**Table 2:** Clinical evaluations on admission and at 1-, 6- and 12-month follow-up

		**Admission**	**Hospital discharge**	**1 month**	**6 month**	**12 month**
**Demographic, clinical and functional characteristics, and key time segments**						
Demographic variables						
	Race, gender, education	X				
	Marital and employment status	X	X	X	X	X
Physical examination (weight, height, blood pressure, heart rate and rhythm)		X				
Laboratory values (serum creatinine, hemoglobin, fasting lipid profile)		X				
Etiology of ALI-CLI		X				
Key time segments (see ‘List of key time segments’)		X	X			
Length of hospital stay			X			
Endovascular and open surgical procedures			X			
Ambulatory status		X	X	X	X	X
**Risk factors and risk-reduction therapies**						
Historical risk factors		X				
Use of risk-reduction pharmacotherapies		X	X	X	X	X
Smoking status		X	X	X	X	X
**Outcome events**						
Amputation (major or minor)			X	X	X	X
Cardiovascular ischemic events (non-fatal MI and stroke), death			X	X	X	X
Recurrence of ALI-CLI			X	X	X	X
Other causes of hospitalization				X	X	X

“Minor amputation” is defined as removal of any part of the lower limb below the ankle and “major amputation” is defined as removal of part of the lower limb above the ankle. All-cause death is ascertained via the social security death index (SSDI) in the USA or via other national death registry systems using a combination of the social security number (SSN), name and birth date to identify cases. Searching the online SSDI, with a correct SSN provides an accurate method to determine death as an outcome in clinical research studies. A majority of subjects ordinarily lost to followup including those with sudden death outside the hospital can thus have their vital status accurately determined [16]. Cardiovascular death includes fatal stroke, fatal MI, and other cardiovascular death. Other cardiovascular death includes deaths of cardiac origin: death associated with pulmonary embolism, vascular operation or procedure, amputation (except for trauma or malignancy), heart failure and visceral or limb infarction. Re-hospitalization for CV events is defined as hospitalization for transient ischemic attack, unstable angina, worsening of PAD, CHF, other ischemic arterial event or bleeding leading to both hospitalization and transfusion. Recurrence of ALI/CLI is defined as occurrence of any second episode of clinically diagnosed ALI/CLI and ascertained by medical chart review at follow-up.

Follow-up will be performed primarily by phone calls at 1 month (with a one week window), 6 months (with a one month window), and 12 months (with a two month window) after hospital discharge using a phone script containing standardized questions. The occurrence of non-fatal MI, non-fatal stroke, amputation, re-hospitalization, recurrence of ALI/CLI are ascertained by medical records, all cause death by social security death index website [17], and CV death by medical record review. Ambulatory status will be ascertained by self-report during follow-up calls and at return visits.

### Determination of sample size

This is a hypothesis-generating registry from which preliminary data will be obtained that will be key to refining values used for estimating statistical power for future multicenter clinical trials. It is anticipated that these data may be useful in identifying medical, endovascular, and health system interventions that could be tested in a future randomized clinical trial, providing more accurate estimates of expected detectable differences and their standard deviations. Therefore, a formal power analysis was not undertaken in the design of this pilot registry.

### Statistical analysis

Descriptive statistics will be presented as means and standard deviations for continuous variables, and frequencies and percentages for categorical data. Continuous data that are not normally distributed will be analyzed by nonparametric methods. Key time variables will be analyzed as continuous variables, and also categorized to allow assessment of dose–response patterns. Univariate models for all outcome variables with demographic, clinical and risk factor variables will be used to elucidate potential confounding variables. Multivariable logistic regression models will be used to assess the association between key time variables and short-term binary outcomes (amputation, MI, stroke and death), with adjustment for confounders. Survival analysis will be performed using Kaplan-Meier methods for preliminary analyses, and Cox proportional hazards models with adjustment for confounders. Multiple linear regression models will be used to assess the associations between time segment variables and length of stay, with adjustment for confounders. A two-sided p-value of <0.05 will be considered statistically significant. Data analysis will be performed using Stata (Stata Inc, College Station, TX, USA).

## Discussion

It is well-known that patients presenting with advanced PAD bear a high burden of systemic ischemic events and suffer an adverse limb prognosis, with very high health economic impact [18]. Thus, care for patients with advanced PAD is a critical public health issue in the USA and internationally [19]. Current epidemiological data suggest that the incidence of ALI is 130 to 160 per million per year [3,7] and the incidence of CLI is approximately 340 to 1000 per million per year [2,4]. Major amputation rates at 30 days after clinical presentation have been reported to be 10-30%, and death at one year is reported to range from 16-42% for ALI patients [7,20]. Patients with CLI face a comparably dismal prognosis and the annual amputation rate has been reported to vary between 10% and 40% after a first diagnosis of CLI [21]. Overall, one- and five-year mortality rates of patients with CLI are reported to be 20-25% and 40-70%, respectively [22-24]. There are few cardiovascular diseases with such adverse short-term outcomes. However, diseases with high rates of adverse short term outcomes are favorable for study within a disease registry frame.

The FRIENDS Registry was designed to enroll a high volume of sequential patients with advanced PAD in order to collect an adequate sample to permit evaluation of the contributions of patient demographics, PAD etiology (e.g., cardioembolism vs. graft failure), key time segments, use of risk-reduction medications, and revascularization strategies on functional status, rates of revascularization, amputation, systemic ischemic event rates, and death. These data will also provide the opportunity to determine how clinicians in practice assess patients with advanced PAD in real world (not clinical trial) settings, including data that might define rates of adherence to current evidence-based clinical care guidelines.

We hypothesize that the current arbitrary 14 day diagnostic “time-based syndrome definition” that is often used to differentiate ALI and CLI (beyond use of the 6 “Ps”) may not adequately discriminate these two syndromes. The rationale for use of the 14-day timeframe to define ALI has not been based on evidence as robust as the data used to define the efficacy of acute MI or stroke treatments. While there was a biological rationale for setting an objective 14-day, time-based inclusion criteria for study of catheter-directed thrombolysis in individuals with acute arterial occlusion [20,25], this time-based definition has been carried forward into subsequent PAD care guidelines without corroborating the utility of this approach for endovascular and open surgical techniques.

This registry provides an opportunity to assess whether ALI or CLI are indeed two distinct clinical syndromes with different etiologies and outcomes, or whether these traditional clinical distinctions alternatively represent a time-dependent continuum of advanced PAD.

More than 20 years ago, Panetta et al. [26] observed that a longer duration of ischemia was associated with increased amputation rates in ALI patients, and a more recent study suggested that an increased duration of limb ischemia was associated with more extensive gangrene [27]. Nevertheless, these observations were derived from small cohorts, did not clarify a precise vulnerable period, nor elucidate other factors that might affect limb outcomes. This registry may provide insights into factors that, along with prolonged pre-admission limb ischemia, worsen prospects for limb survival. The registry will also provide a contemporary description of current etiologies of limb ischemia. For example, does atrial fibrillation contribute to limb ischemic events at a rate documented a decade ago, now that antithrombotic therapies are more widely used? Is graft surveillance used, per current national care guidelines, at rates adequate to protect individuals who have undergone prior successful limb revascularization?

During the last decade, cardiovascular care guidelines have become an important cornerstone of the management of heart disease and the positive impact of guidelines is known [28]. In contrast, the development and dissemination of PAD care guidelines, including recommendations regarding the ideal management of ALI and CLI, are rarely evaluated prospectively [1,2]. These guidelines have recognized that appropriate use of evidence-based risk-reduction medication represents a major opportunity for reducing future myocardial infarction, stroke, and death if used in individuals with recognized PAD. However, recent data have shown poor rates of use of evidence-based risk-reduction medication on admission or discharge in patients with CLI [10,29,30]. The registry will provide an opportunity to evaluate the contemporary use of risk-reduction medications to prevent these major ischemic events in the PAD population at highest short-term risk. We hypothesize that higher use of these medications would offer a potentially major contribution to improvement in rates of amputation-free survival; only a prospective registry with an adequate cohort sample is likely to provide such data, as a prospective RCT to evaluate this hypothesis is currently unlikely. Data further demonstrating such benefits of risk-reduction medication use might then be considered for future guideline inclusion, or within future PAD performance standards [31].

Currently there are no standardized quality-of-life instruments that are widely applied in populations of individuals with critical limb ischemia. This registry offers an opportunity to evaluate functional status on admission, at discharge and at follow-up, as well as other predictors of functional status at follow-up. While it is known that poor ambulatory status on presentation is associated with loss of primary patency and higher rates of amputation at six months follow-up, other factors beyond patency (e.g., avoidance of readmission, social support, etc.) may contribute to the achievement of improved functional outcomes [32].

Several limitations are inherent to this (or any) registry. First, the enrollment of patients is intended to be consecutive, but this is never fully achieved for any registry. Nevertheless, the majority of the patients who present for hospital care may be enrolled if, as planned, both hospitalists and vascular specialists are engaged at registry launch. Second, as for any registry, selection for inclusion of a wide array of hospitals and health systems can facilitate accrual of a representative cohort. Third, any PAD registry is encumbered – in contrast to CAD or stroke registries – by the inconsistent use of objective diagnostic criteria in real world clinical practice (in contrast to RCTs). Whereas all individuals with an acute MI undergo an ECG, it is not now clear whether individuals with advanced PAD undergo consistent vascular laboratory testing. Thus, we have elected to initially permit the inclusion of all “clinically diagnosed” ALI and CLI without the requirement of pedal or toe pressure measurements. Analysis of cohorts who have, or have not, undergone such testing can demonstrate the utility of this diagnostic approach. We note that the past diagnostic inclusion criteria of an ankle pressure of ≤50 mmHg [33] would potentially exclude two-thirds of patients with CLI, as only 30% would fulfill the European Consensus Guideline pedal pressure criteria, as observed in the BASIL trial [34]. Thus, the registry will provide valuable information in a “real-world” setting by using broader inclusion criteria. Pre hoc defined subgroup analysis of the “clinical diagnosis” and “objective diagnosis” groups will provide insight regarding the value of current diagnostic pathways on choice of therapy and achievement of positive outcomes.

In conclusion, a multicenter advanced PAD registry may represent a significant opportunity to assess a series of hypothesis-based factors that may modify systemic or limb outcomes. In a manner analogous to methods used to better understand the natural history and impact of treatments for acute MI, stroke and heart failure, advanced PAD registry data that includes pre-hospital factors, revascularization strategies used during an index hospitalization, and that evaluates long-term use of risk modifying pharmacotherapies may provide new insights to help the design of future PAD clinical trials.

### Trial status

Recruitment for a multi-center CLI and ALI registry began in February 2007 at a single center and will continue at other centers, dependent upon funding.

## Abbreviations

ABI: Ankle-brachial index; ACS: Acute coronary syndromes; ALI: Acute limb ischemia BMI, body mass index; CAD: Coronary artery disease; CLI: Critical limb ischemia; CV: Cardiovascular; CVD: Cerebrovascular disease; MI: Myocardial infarction; NCEP: National Cholesterol Education Program; PAD: Peripheral artery disease; RCTs: Randomized clinical trials; TBI: Toe-brachial index; TIA: Transient ischemic attack; SSDI: Social security death index; SSN: Social security number.

## Competing interests

The authors have no financial or non-financial competing interests.

## Authors’ contributions

HHK, SD and ATH made substantial contributions to study concept and design; ATH and HHK acquired funding for the study; IB, NCO, MRJ, JG, JMP, AST, TDH, RVL participated in its design and helped to draft the manuscript. All authors read and approved the final manuscript.

## Pre-publication history

The pre-publication history for this paper can be accessed here:

http://www.biomedcentral.com/1471-2261/13/120/prepub
